# Supplementary material for: Identification of Host Factors Interacting with a γ-Shaped RNA Element from a Plant Virus-Associated Satellite RNA
Source: Viruses. 2023 Oct 1;15(10):2039. doi: 10.3390/v15102039 (PMC10611174; doi:10.3390/v15102039)
Supplement: Supplementary file 1 [file viruses-15-02039-s001.zip › Supplementary Figures-R1.pptx]

## Slide 1
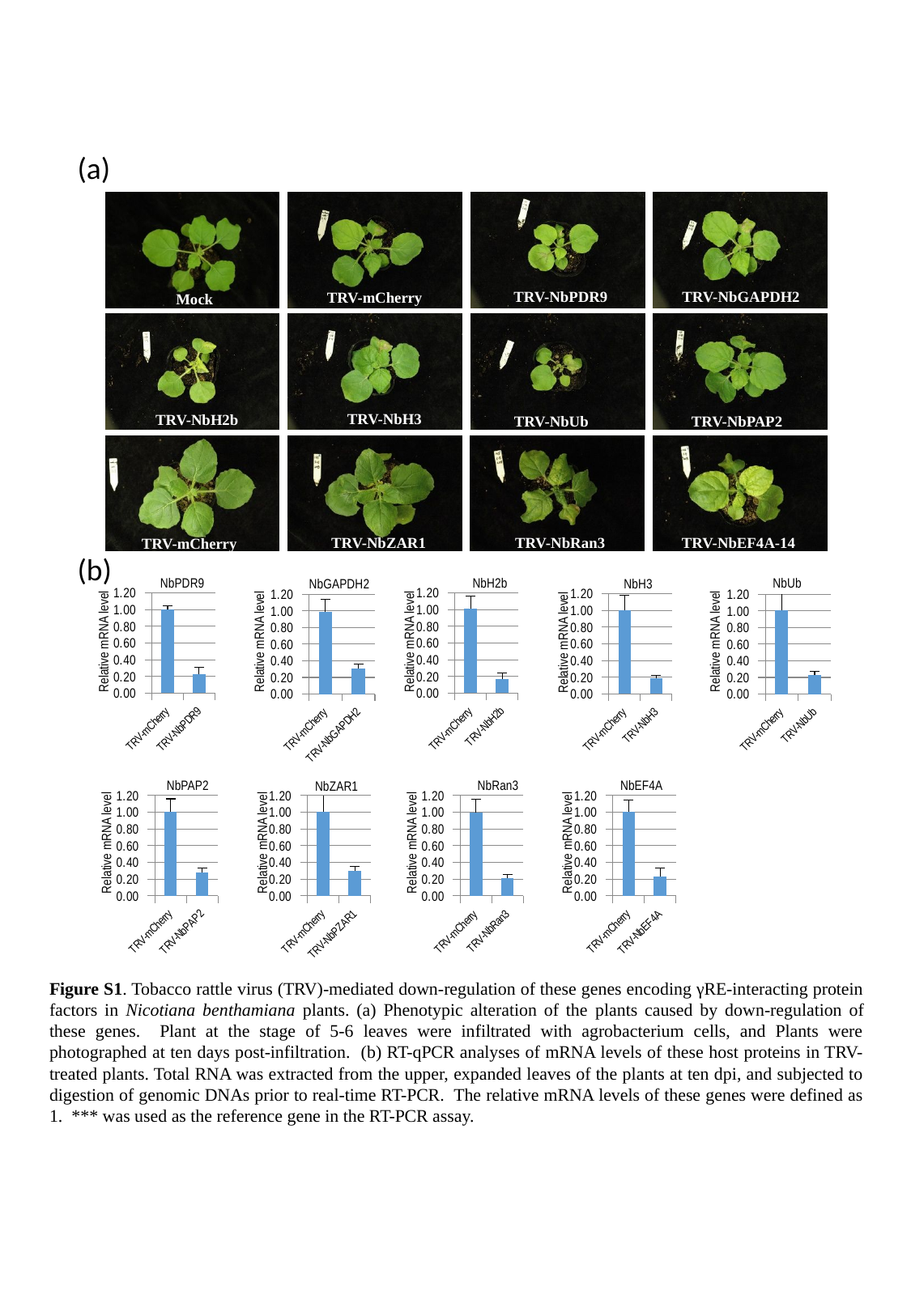

(a)
TRV-NbPDR9
TRV-NbGAPDH2
TRV-mCherry
Mock
TRV-NbH3
TRV-NbH2b
TRV-NbUb
TRV-NbPAP2
TRV-NbZAR1
TRV-NbRan3
TRV-NbEF4A-14
TRV-mCherry
(b)
NbPDR9
NbH2b
NbUb
NbH3
NbGAPDH2
### Chart
| Category | |
|---|---|
| TRV-mCherry | 1.0 |
| TRV-NbPDR9 | 0.22666666666666666 |
### Chart
| Category | |
|---|---|
| TRV-mCherry | 1.01 |
| TRV-NbH2b | 0.17 |
### Chart
| Category | |
|---|---|
| TRV-mCherry | 1.0 |
| TRV-NbH3 | 0.18666666666666668 |
### Chart
| Category | |
|---|---|
| TRV-mCherry | 0.9899999999999999 |
| TRV-NbGAPDH2 | 0.30333333333333334 |
### Chart
| Category | |
|---|---|
| TRV-mCherry | 1.0033333333333332 |
| TRV-NbUb | 0.22666666666666666 |Relative mRNA level
Relative mRNA level
Relative mRNA level
Relative mRNA level
Relative mRNA level
NbPAP2
NbRan3
NbEF4A
NbZAR1
### Chart
| Category | |
|---|---|
| TRV-mCherry | 1.01 |
| TRV-NbPAP2 | 0.2833333333333334 |
### Chart
| Category | |
|---|---|
| TRV-mCherry | 1.01 |
| TRV-NbPZAR1 | 0.3 |
### Chart
| Category | |
|---|---|
| TRV-mCherry | 0.9933333333333333 |
| TRV-NbRan3 | 0.21666666666666665 |
### Chart
| Category | |
|---|---|
| TRV-mCherry | 1.0066666666666668 |
| TRV-NbEF4A | 0.2333333333333333 |Relative mRNA level
Relative mRNA level
Relative mRNA level
Relative mRNA level
Figure S1. Tobacco rattle virus (TRV)-mediated down-regulation of these genes encoding γRE-interacting protein factors in Nicotiana benthamiana plants. (a) Phenotypic alteration of the plants caused by down-regulation of these genes. Plant at the stage of 5-6 leaves were infiltrated with agrobacterium cells, and Plants were photographed at ten days post-infiltration. (b) RT-qPCR analyses of mRNA levels of these host proteins in TRV-treated plants. Total RNA was extracted from the upper, expanded leaves of the plants at ten dpi, and subjected to digestion of genomic DNAs prior to real-time RT-PCR. The relative mRNA levels of these genes were defined as 1. *** was used as the reference gene in the RT-PCR assay.

## Slide 2
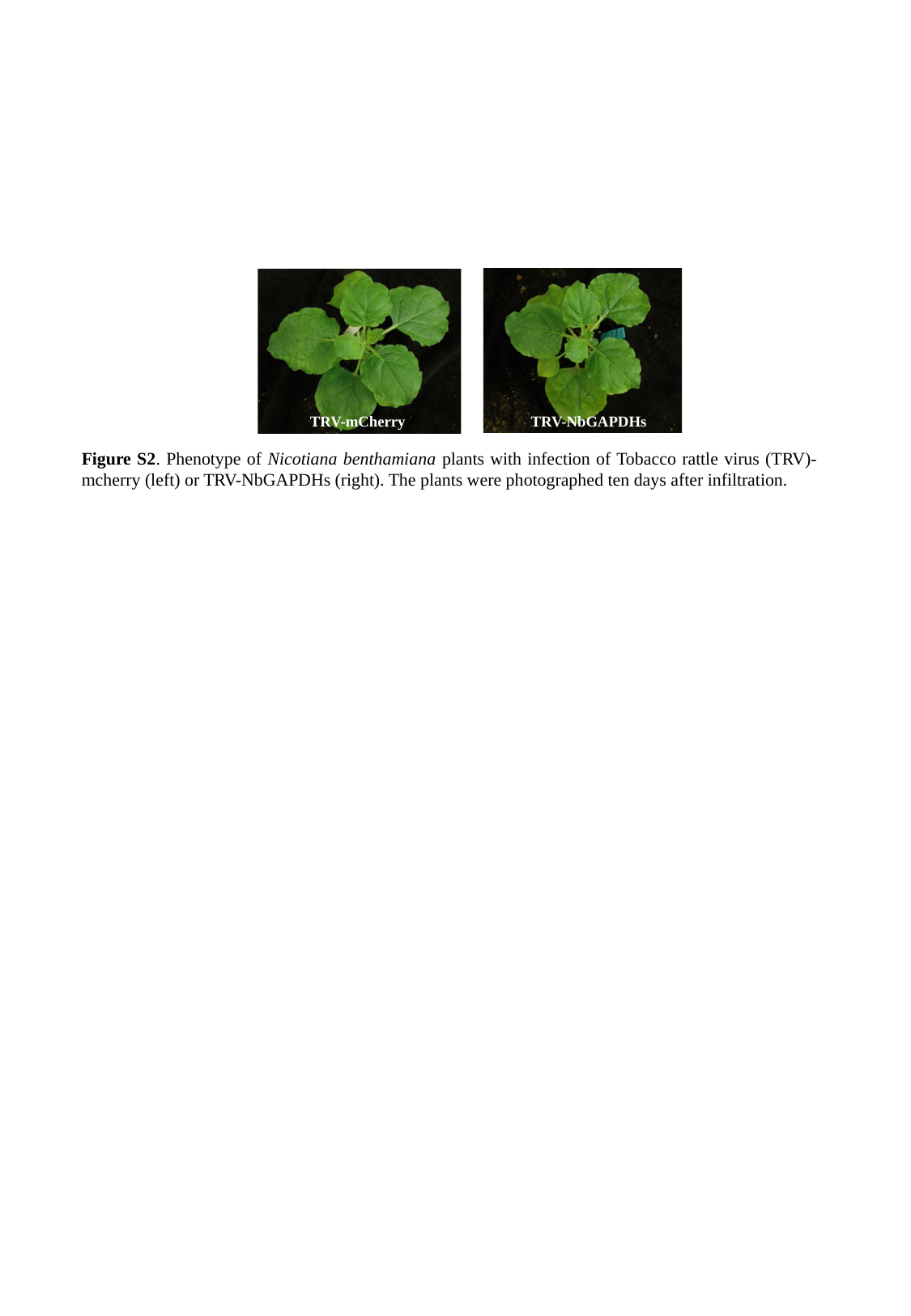

TRV-NbGAPDHs
TRV-mCherry
Figure S2. Phenotype of Nicotiana benthamiana plants with infection of Tobacco rattle virus (TRV)-mcherry (left) or TRV-NbGAPDHs (right). The plants were photographed ten days after infiltration.
