# Supplementary material for: Identification of Host Factors Interacting with a γ-Shaped RNA Element from a Plant Virus-Associated Satellite RNA
Source: Viruses. 2023 Oct 1;15(10):2039. doi: 10.3390/v15102039 (PMC10611174; doi:10.3390/v15102039)
Supplement: Supplementary file 1 [file viruses-15-02039-s001.zip › Table S1 & S2-oligos-R1.pdf]

Table S1 Primers used to amply the DNA fragments of host proteins

Gene name	Primer name	Primer sequence (5'-3')*
NbPDR9	PDR9-F	CGggatccAGCAACATTTTTCCTGGA
	PDR9-R	TCCcccgggGGGATCTCAGGTGGT
NbGAPDH1	GAPDH1-F	TATggatccGGAAAAAAGTAAAATTTCATTCATGACA
	GAPDH1-R	AAAcccgggCTGCCATATGGCTTCTG
NbGAPDH2	GAPDH2-F	CGggatccACATCCCTAAATCACATCAAAG
	GAPDH2-R	TCCcccgggCAGACTTCGTGGGAGA
NbGAPDH3	GAPDH3-F	AAAggatccATTATACACTGATAGCAAAACAAATCA
	GAPDH3-R	AAAcccgggCCTTAGCGTGGTGGT
NbH2b	H2b-F	CGggatccTCCCGAGAAGTGATAGTAG
	H2b-R	TCCcccgggTGAGAAGGCACCAGT
NbZAR1	ZAR1-F	CGggatccAATGTCAGAACGCAAGAG
	ZAR1-R	TCCcccgggCGAGCACTGCTAACAAC
NbH3	H3-F	CGggatccCTCCTAGCGAGCTGAAT
	H3-R	TCCcccgggCACTAAGGCTGCGAG
NbRan3	Ran3-F	CGggatccCCGCAAAGAACAATGGG
	Ran3-R	TCCcccgggCGATGGTGGAACTGG
NbeIF4A	eIF4A-F	CGggatccAGGTAGTTCTCTGGCTG
	eIF4A-R	TCCcccgggTAATGTTGACAAGGAAGAGTG
NbUb	Ub-F	CGggatccTTTTTTGACAGAAATCGGAATC
	Ub-R	TCCcccgggGGTATCCCACCCGA
NbPAP2	PAP2-F	CGggatccCTTTAGGTCTTCTTCGCTC
	PAP2-R	TCCcccgggGGCTTCTCTACTTCAGTAC

* "ggatcc": BamHI site, "cccggg": SmaI site

Table S2 Primers used to quantify mRNA levels in real-time RT-PCR reactions

Gene name	Primer name	Primer sequence (5'-3')*
NbPDR9	PDR9-qF	GCAAAGGTCCATGTAGGA
	PDR9-qR	TAGAGTCAACCTTGAAGG
NbGAPDH2	GAPDH2-qF	AAGCAGTTCAGAATAAAGTAC
	GAPDH2-qR	CATACAAACACAAGAACC
NbH2b	H2b-qF	GAAGCCTACTATCACTTCTC
	H2b-qR	CACAAACTCTAAAATTGATTA
NbZAR1	ZAR1-qF	ATCCATTCCGACATTGCC
	ZAR1-qR	GCTGAAATCCAAAATCTGTA
NbH3	H3-qF	AAAGCAGTGCTGTTGCTG
	H3-qR	CTTTCACCACGAATCCTC
NbRan3	Ran3-qF	GGAAGAAGAATCTGCAATAC
	Ran3-qR	GTTGACTGGCAGCAGCAG
NbeIF4A	eIF4A-qF	ACTCTTTGCGATCTTTACGA
	eIF4A-qR	TCAGTGGTGATGAGCACA
NbUb	Ub-qF	CCTTCACCTTGTCCTCCGT
	Ub-qR	CATCATTGAGACCTCAGC
NbPAP2	PAP2-qF	CGTCCAGATTAAGTTCGAG
	PAP2-qR	CTCGATATGGTTCTAGCC
